# Prenatal but not continued postpartum vitamin D supplementation reduces maternal bone resorption as measured by C-terminal telopeptide of type 1 collagen without effects on other biomarkers of bone metabolism

**DOI:** 10.1016/j.endmts.2023.100154

**Published:** 2024-03-31

**Authors:** Christine Krupa, Huma Qamar, Karen M. O'Callaghan, Akpevwe Onoyovwi, Abdullah Al Mahmud, Tahmeed Ahmed, Alison D. Gernand, Daniel E. Roth

**Affiliations:** a Centre for Global Child Health, Hospital for Sick Children, Toronto, Canada; b Department of Nutritional Sciences, King's College London, United Kingdom; c Nutrition and Clinical Services Division, International Centre for Diarrhoeal Disease Research, Bangladesh, Dhaka, Bangladesh; d Department of Nutritional Sciences, The Pennsylvania State University, PA, United States; e Department of Nutritional Sciences, University of Toronto, Toronto, Canada; f Department of Paediatrics, Hospital for Sick Children and University of Toronto, Toronto, Canada

**Keywords:** Pregnancy, Postpartum, Vitamin D, Bone turnover, Carboxy terminal telopeptide of type 1 collagen

## Abstract

Vitamin D is a key regulator of bone mineral homeostasis and may modulate maternal bone health during pregnancy and postpartum. Using previously-collected data from the Maternal Vitamin D for Infant Growth (MDIG) trial in Dhaka, Bangladesh, we aimed to investigate the effects of prenatal and postpartum vitamin D_3_ supplementation on circulating biomarkers of bone formation and resorption at delivery and 6 months postpartum. MDIG trial participants were randomized to receive a prenatal;postpartum regimen of placebo or vitamin D_3_ (IU/week) as either 0;0 (Group A), 4200;0 (B), 16,800;0 (C), 28,000;0 (D) or 28,000;28,000 (E) from 17 to 24 weeks' gestation to 6 months postpartum. As this sub-study was not pre-planned, the study sample included MDIG participants who had data for at least 1 biomarker of interest at delivery or 6 months postpartum, with a corresponding baseline measurement (*n* = 690; 53 % of 1300 enrolled trial participants). Biomarkers related to bone turnover were measured in maternal venous blood samples collected at enrolment, delivery, and 6 months postpartum: osteoprotegerin (OPG), osteocalcin (OC), receptor activator nuclear factor kappa-B ligand (RANKL), fibroblast growth factor 23 (FGF23), procollagen type 1 N-terminal propeptide, (P1NP) and carboxy terminal telopeptide of type 1 collagen (CTx). Supplementation effects were expressed as percent differences between each vitamin D group and placebo with 95 % confidence intervals (95 % CI). Of 690 participants, 64 % had 25-hydroxyvitamin D concentrations (25OHD) <30 nmol/L and 94 % had 25OHD < 50 nmol/L at trial enrolment. At delivery, mean CTx concentrations were 27 % lower in group E versus placebo (95 % CI: −38, −13; *P* < 0.001), adjusting for enrolment concentrations. However, at 6 months postpartum, CTx concentrations were not statistically different in group E versus placebo (14 %; 95 % CI: −5.3, 37; *P* = 0.168), adjusting for delivery CTx concentrations. Effects on other biomarkers at delivery or postpartum were not statistically significant. In conclusion, prenatal high-dose vitamin D supplementation reduced bone resorption during pregnancy, albeit by only one biomarker, and without evidence of a sustained effect in the postpartum period. However, further evidence is needed to substantiate potential maternal bone health benefits of vitamin D in the postpartum period.

## Highlights

• Prenatal 28,000 IU vitamin D3/week reduced CTx at delivery, compared to placebo.
• Effect of vitamin D on CTx at delivery mediated by reduction in parathyroid hormone.
• At 6 months postpartum, the effect of high-dose vitamin D on CTx was not sustained.

## 1 Introduction

Osteoporosis is a chronic bone disease characterized by reduced bone strength and a corresponding increased susceptibility to fracture (NIH Consensus Development Panel on Osteoporosis Prevention D and T, 2001). The reduction in quality of life attributed to frailty and musculoskeletal pain places a large burden on healthcare systems, with major economic repercussions (Harvey et al., 2010). In particular, the magnitude of disease prevalence in low- and middle-income countries is increasing due to an ageing population and widespread calcium and vitamin D deficiency (Shlisky et al., 2022). Changes in calcium metabolism and bone remodeling during pregnancy and lactation have potential implications for skeletal integrity in some individuals (Pearson et al., 2004). Vitamin D supplementation may reduce bone resorption or promote bone formation due to its role in the maintenance of calcium and bone-mineral homeostasis (Cranney et al., 2007). However, few trials to date have examined the effects of vitamin D supplementation on maternal bone metabolism during pregnancy or lactation (Wei et al., 2017; Curtis et al., 2021).

The homeorhetic regulation of calcium that occurs during pregnancy facilitates adaptations to meet fetal demands for calcium, although the direct role of vitamin D in maternal-fetal calcium transport is likely minor (Roth and O'Callaghan, 2021). Intestinal calcium absorption is enhanced from early pregnancy, but it is unclear whether this physiological adaptation is directly attributable to the concurrent increase in circulating 1,25-dihydroxyvitamin D (1,25(OH)_2_D), the most biologically active vitamin D metabolite (Kovacs, 2016). Mobilisation of maternal calcium stores is reflected by an increase in bone resorption and subsequent reduction in bone mineral density (BMD) and bone mineral content (BMC), with the hip and lumbar spine considered most susceptible (Møller et al., 2012; Olausson et al., 2008). Although bone loss occurs throughout pregnancy and persists into lactation irrespective of calcium intake (Olausson et al., 2008; Diogenes et al., 2021), observational studies have demonstrated a complete recovery of bone mass in the majority of women within the first 1–2 years postpartum (Pearson et al., 2004; Møller et al., 2012; Diogenes et al., 2021). However, few studies to date have been conducted among populations with a high prevalence of vitamin D deficiency (Wei et al., 2017; Curtis et al., 2021), where targeted interventions that increase maternal 25-hydroxyvitamin D (25OHD, the biomarker of vitamin D status) may support the maintenance of a net balance of bone formation and resorption (Curtis et al., 2021; Park et al., 2017; Haliloglu et al., 2011).

Biomarkers of bone turnover are broadly categorized as markers of bone formation or resorption (Wheater et al., 2013). Osteocalcin (OC), osteoprotegerin (OPG) and procollagen type 1 amino-terminal propeptide (P1NP) represent bone formation activity (Wheater et al., 2013), whereas receptor activator nuclear factor kappa-B ligand (RANKL) and carboxy terminal telopeptide of type 1 collagen (CTx) represent bone resorption (Wheater et al., 2013). Much attention has been given to fibroblast growth factor 23 (FGF23) as a biomarker of bone mineral metabolism due to its role in the regulation of phosphate and vitamin D homeostasis (Sirikul et al., 2022). Additionally, parathyroid hormone (PTH) secretion increases in response to low vitamin D status, which stimulates bone resorption to maintain serum calcium levels (Khundmiri et al., 2016). Observational studies have shown inverse associations between circulating 25OHD and biomarkers of bone turnover during pregnancy and postpartum, suggesting maternal vitamin D deficiency exacerbates bone resorption (Park et al., 2017; Haliloglu et al., 2011). Trial data from the UK support these findings, in which a lower rise in urinary CTx throughout gestation was reported following intervention with vitamin D supplementation (Curtis et al., 2021). However, in a trial in the US, there was no effect of prenatal vitamin D supplementation on the change in maternal BMD and BMC between 12-20 weeks of gestation and 0-14 weeks postpartum (Wei et al., 2017).

Although maternal vitamin D supplementation is a feasible strategy to increase maternal and neonatal 25OHD concentrations (Roth et al., 2017), it is unclear whether an improvement in vitamin D status suppresses maternal bone resorption among populations accustomed to a habitually low vitamin D status. Using data and biological samples from a previously-reported randomized, placebo-controlled trial in Dhaka, Bangladesh, the aim of the present study was to examine the dose-ranging effects of prenatal with or without postpartum vitamin D supplementation on circulating markers of bone formation (OPG, OC and P1NP), bone resorption (RANKL and CTx) and bone mineral metabolism (FGF23), in women at delivery and 6 months postpartum. As a previous analysis in this trial showed vitamin D supplementation suppressed intact parathyroid hormone (iPTH) concentrations in a dose-dependent manner (Roth et al., 2018), we further examined whether effects on circulating iPTH mediated observed effects of the vitamin D intervention on bone biomarkers.

## 2 Materials and methods

### 2.1 Data source

This sub-study is based on the secondary use of data and samples from the Maternal Vitamin D for Infant Growth (MDIG) trial, for which the methods and primary trial findings were previously described (Roth et al., 2018; Roth et al., 2015). Briefly, MDIG was a randomized, placebo-controlled, dose-ranging trial of maternal prenatal and postpartum vitamin D supplementation in Dhaka, Bangladesh (clinicaltrials.gov identifier: NCT01924013). A total of 1300 women with uncomplicated singleton pregnancies were recruited at 17–24 weeks of gestation and randomized to receive a prenatal; postpartum regimen of 0;0 (Group A), 4200;0 (Group B), 16,800;0 (Group C), 28,000;0 (Group D) or 28,000;28,000 (Group E) IU cholecalciferol (vitamin D_3_)/week until 6 months postpartum. All women were provided with daily iron-folic acid supplementation in line with routine antenatal care, in addition to 500 mg/d calcium (as calcium carbonate) (Roth et al., 2015) to attenuate competing effects of low dietary calcium intakes (Heaney, 2007). Vitamin D/placebo tablets were administered under direct observation by study personnel, and adherence to the prenatal and postpartum vitamin D intervention was calculated as the proportion of scheduled doses that were received. Adherence to the calcium co-intervention was not routinely assessed but was expected to be similar across trial arms given the randomized design. Written informed consent was obtained from all participants prior to commencing the trial, including consent for future use of stored data and biological samples. The MDIG trial was approved by research ethics committees at the Hospital for Sick Children, Toronto, Canada (REB#1000039072) and the International Centre for Diarrheal Disease Research, Bangladesh (icddr,b) (PR-13055), and additional approval was obtained for the secondary use of trial data and stored samples in this sub-study at the Hospital for Sick Children (REB#1000060519). Relevant health and socio-demographic characteristics were recorded at enrollment (17–24 weeks' gestation) by structured interviews using standardized data collection forms. Relative asset index was determined by ownership of household items, derived using principal components analysis (Roth et al., 2015). Non-fasting venous blood samples were collected and processed to serum or plasma according to standard procedures and then stored at ≤−70 °C and transported on dry ice prior to analysis.

### 2.2 Participant and sample selection

**Fig. 1:**
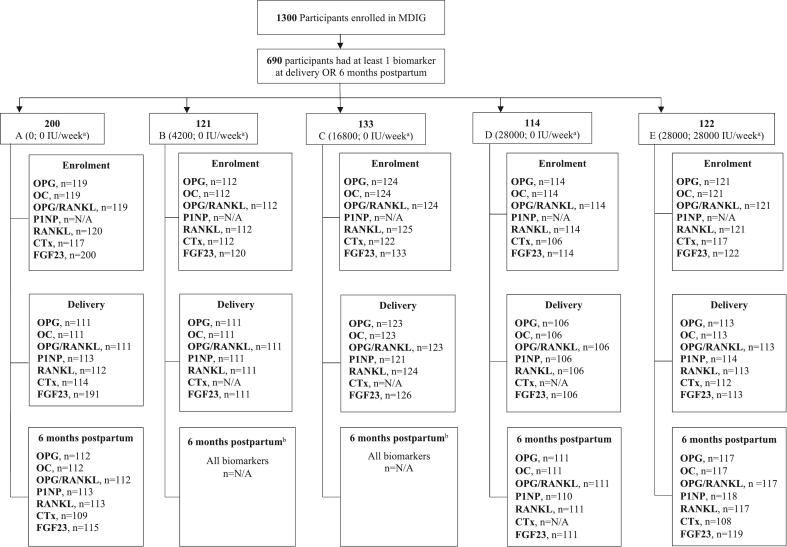
Participant selection flow diagram. Selection reflects the primary analysis of the study. ^a^Intervention regimens in each group shown as the “prenatal; postpartum” vitamin D (or placebo) dose assigned at randomization. ^b^Groups B and C: OPG, OC, RANKL, P1NP and CTx samples at 6 months postpartum were not analysed (cost saving measure), so these groups were excluded from analyses at the 6-month postpartum timepoint.

Data and samples from MDIG trial participants were included in the present analysis if the serum or plasma concentration of at least one bone biomarker of interest was quantified at delivery or 6 months postpartum, with a corresponding baseline (enrolment) measurement to facilitate adjustment of baseline concentrations in statistical analyses (Fig. 1). As a cost-saving measure, enrolment samples were not initially analysed for P1NP or CTx, and only participants from groups A, D and E were selected for 6-month postpartum analyses (with the exception of FGF23, for which postpartum data had already been generated) (Roth et al., 2015). As groups D and E received identical prenatal (28,000 IU/week) but different postpartum (0 vs 28,000 IU/week, respectively) vitamin D doses, group D was included in the postpartum analyses to enable comparisons of the prenatal-only to the combined prenatal and postpartum (28,000; 28,000 IU/week) vitamin D intervention. Based on initial observations of changes in CTx in the prenatal period, a post-hoc decision was made to expand the CTx analyses to include enrolment samples from all intervention groups.

### 2.3 Laboratory analyses

All assays were performed at the Analytical Facility for Bioactive Molecules (AFBM; The Hospital for Sick Children, Toronto), except for CTx and P1NP assays which were performed by MDIG team personnel at The Hospital for Sick Children, Toronto (Supplemental Table 1). Plasma OPG, OC and RANKL concentrations were analysed using multiplex immunoassays. Intra- and inter-assay CVs for OPG, OC and RANKL were all <10 %. (Supplemental Table 1). Serum intact P1NP, and plasma CTx, iPTH and FGF23 (c-terminal) concentrations were measured using commercial Enzyme-Linked Immunosorbent Assay (ELISA) kits (Supplemental Table 1). Intra-assay CVs for CTx and iPTH were ≤10 % and inter-assay CVs were <20 %; both intra- and inter-assay CVs for FGF-23 were <10 %. Analysis of CTx followed a staged approach in batches determined by treatment group and timing of sample collection (Supplemental Methods). Analysis of serum 25OHD was conducted at AFBM by high performance liquid chromatography-tandem mass spectrometry, as described previously (Roth et al., 2018). This laboratory participated in the Vitamin D External Quality Assessment Scheme (DEQAS; Charing Cross Hospital, London, UK). For all assays, values that were below the lower limit of quantification (LOQ) were imputed as half the lower LOQ and those that were above the upper LOQ were imputed as the value of the upper LOQ (Supplemental Table 1).

### 2.4 Statistical analysis

Distributions were assessed using histograms and boxplots. Biologically implausible high values were identified for OC concentrations only (*n* = 2) and removed from further analyses. Due to right skewed distributions, all biomarkers were natural log-transformed before analyses to approximate normality, and geometric means and 95 % confidence intervals (95 % CI) were used to summarize each bone biomarker by group, at each time point. Differences in participant characteristics across groups were tested by Chi-square and ANOVA or Kruskal-Wallis tests, as appropriate. As inclusion in the present study was dependent on measured biomarker availability, differences were explored between participants of the present study and MDIG trial participants who were not included in this sub-study, using independent samples *t*-tests and Chi-square tests for continuous and categorical variables, respectively.

#### 2.4.1 Effect of vitamin D supplementation on bone biomarkers at delivery and postpartum

To estimate the effect of vitamin D supplementation on bone biomarkers at delivery and postpartum, linear regression models were fitted using vitamin D intervention group as the categorical exposure variable (with placebo as the reference group) and the biomarker of interest as the continuous outcome variable. With the exception of analyses involving CTx, groups D and E were combined to create a high-dose (28,000 IU/week) prenatal vitamin D supplementation group but were disaggregated in postpartum analyses to test effects of the prenatal only versus prenatal plus postpartum intervention. Except for P1NP, for which data at enrollment were not available, all models included adjustment for the corresponding biomarker concentration at enrollment. As a post-hoc analysis, where the effect of the vitamin D intervention at delivery was significant (*P* < 0.05), regression models testing the intervention effect on the biomarker at 6 months postpartum instead included adjustment for delivery biomarker concentrations, which was interpreted as the postpartum effect. A Tobit regression model was fitted for FGF23 at delivery to account for right censoring of the distribution due to a large proportion of values that were above the upper LOQ of the assay (Supplemental Table 1). Tobit regression models were also used for postpartum P1NP and delivery and postpartum RANKL to account for left-censoring of the distributions due to high proportions of values below the lower LOQ (Supplemental Table 1). Effects were expressed as mean percent differences and 95 % CIs for comparison of the biomarker concentration in each vitamin D group relative to placebo. Differences were considered statistically significant if *P* < 0.05. Formal correction for multiple testing was not employed.

Primary analyses included all participants, irrespective of adherence to the intervention. Per-protocol sensitivity analyses were limited to participants who consumed ≥90 % of the assigned trial supplements and did not consume non-study vitamin D/calcium supplements (Supplemental Fig. 1). In further sensitivity analyses, we excluded outliers based on visual inspection of boxplots, stratified by intervention group. Post-hoc analyses were also conducted to account for between-batch variation in CTx concentrations (Supplemental Methods, Supplemental Figs. 2, 3).

#### 2.4.2 Mediation analysis investigating the contribution of parathyroid hormone to the effects of vitamin D supplementation on bone biomarkers

Following the approach by Baron and Kenny (Baron and Kenny, 1986), and where effects of primary analyses were significant (*P* < 0.05), we tested whether iPTH mediated the effect of the vitamin D intervention on the bone biomarker of interest. This approach involved three groups of linear regression models: (i) regression of the bone biomarker on vitamin D intervention group to estimate total effects; (ii) regression of iPTH on the vitamin D intervention group and regression of the bone biomarker on iPTH to estimate indirect effects; and (iii) regression of the bone biomarker on the vitamin D intervention group and iPTH to estimate the direct effect. We interpreted effects to be mediated, at least in part, by iPTH if the direct effect of vitamin D intervention on the bone biomarker obtained from model iii attenuated the total effect from model i such that the total effect was no longer significant. In a post-hoc analysis, a similar mediation model was constructed using 25OHD at delivery (rather than the vitamin D intervention group) as the exposure variable to test the total and indirect effects of achieved 25OHD on the bone biomarker of interest following supplementation with vitamin D.

#### 2.4.3 Post hoc analysis by timing of CTx sample collection

In a post-hoc analysis, we used linear regression to examine if there were any differences between intervention groups in the timing of CTx sample collection, given that samples were collected in a non-fasting state and CTx has been shown to exhibit circadian variation (Qvist et al., 2002).

All analyses were performed using Stata v16.1 (StataCorp, College Station, TX).

## 3 Results

### 3.1 Participant characteristics

In total, 690 participants met eligibility criteria for this sub-study but analytical sample sizes differed by biomarker and time point (enrollment, delivery, 6 months postpartum). Consistent with previous findings from the MDIG trial (Roth et al., 2018), 64 % of women were vitamin D deficient prior to intervention, using <30 nmol/L as recommended by the Institute of Medicine (Institute of Medicine, 2011), and almost all (94 %, *n* = 643) had 25OHD concentrations <50 nmol/L. As previously reported (Roth et al., 2018), there was a significant effect of vitamin D supplementation on 25OHD across treatment groups at delivery (*p* < 0.001) and 6 months postpartum (p < 0.001); median serum 25OHD was >50 nmol/L in groups B, C, D and E at delivery, and at 6 months postpartum remained higher in group E compared to all other groups (Table 1). Participant characteristics were otherwise similar across trial arms with the exception of minor differences in the distributions of height and gestational duration (Table 1). Participants in this sub-study were generally comparable to other MDIG trial participants, although women in the present study were more likely to give birth by C-section (Supplemental Table 2). Biomarker data were equally available across intervention groups at both delivery and postpartum time points (Table 2).

**Table 1:** Participant characteristics in a pregnancy cohort in Dhaka, Bangladesh, by intervention group.

Characteristic^a^	Intervention group (prenatal;postpartum vitamin D dose, IU/week)					
	Placebo (0;0)	B (4200;0)	C (16,800;0)	D (28,000;0)	E (28,000;28,000)	*P*^b^
Number of participants^c^	200	121	133	114	122	
Gestational age at enrollment	20 (19, 22)	21 (19, 22)	20 (19, 22)	20 (19, 22)	21 (19, 22)	0.63
Age (years)	22 (20, 26)	22 (20, 25)	22 (20, 25)	22 (20, 27)	23 (20, 27)	0.78
Height (cm)	151.2 (5.4)	150.9 (5.3)	150.8 (5.8)	149.7 (5.9)	152.0 (5.4)	0.03
BMI at enrollment (kg/m^2^)^d^	24.0 (4.2)	23.3 (3.9)	23.7 (4.1)	23.6 (3.4)	23.8 (4.0)	0.69
Asset index, n (%)						0.12
1 (lowest)	44 (22)	30 (25)	19 (14)	22 (19)	22 (18)	
2	36 (18)	26 (22)	32 (24)	16 (14)	17 (14)	
3	47 (24)	18 (15)	21 (16)	24 (21)	22 (18)	
4	34 (17)	20 (17)	29 (22)	31 (27)	34 (28)	
5 (highest)	39 (20)	26 (22)	32 (24)	20 (18)	27 (22)	
Education, n (%)						0.26
No education	4 (2.0)	6 (5.0)	9 (6.8)	4 (3.5)	3 (2.5)	
Primary incomplete	39 (20)	26 (21)	21 (16)	21 (18)	26 (21)	
Primary complete	28 (14)	20 (17)	10 (7.5)	18 (16)	21 (17)	
Secondary incomplete	86 (43)	36 (30)	60 (45)	43 (38)	45 (37)	
Secondary complete	43 (22)	33 (27)	33 (25)	28 (25)	27 (22)	
Occupation, n (%)						0.93
Unemployed	189 (95)	113 (93)	125 (94)	107 (94)	117 (96)	
Employed	11 (5.5)	8 (6.6)	8 (6)	7 (6.1)	5 (4.1)	
Serum 25OHD at enrollment (nmol/L)^d^	26.2 (17.4, 35.2)	25.2 (16.3, 32.8)	25.0 (17.7, 35.8)	23.9 (16.2, 34.2)	24.7 (17.4, 33.4)	0.54
Secondary hyperparathyroidism, n (%)^e^	25 (13)	9 (7.5)	12 (9.1)	16 (14)	15 (12)	0.43
Serum 25OHD at delivery (nmol/L)	21.8 (14.2, 29.5)	66.0 (54.7, 77.6)	101.5 (82.6, 116.3)	110.1 (92.9, 128.5)	111.1 (94.3, 132.7)	<0.001
Serum 25OHD at 6 months postpartum (nmol/L)	27.6 (20.1, 39.2)	29.0 (21.7, 37.4)	44.8 (36.3, 51.4)	50.9 (43.9, 58.2)	101.1 (90.9, 117.6)	<0.001
Gestational duration (weeks)	39 (38, 40)	39 (38, 40)	39 (38, 40)	39 (38, 40)	39 (39, 40)	0.04
Exclusive breastfeeding duration (weeks)^f^	13 (3.75, 21)	12 (6, 19)	13 (4, 22)	13 (5.5, 23)	14.5 (4.5, 22)	0.86

a Data presented as median (25th percentile, 75th percentile) or mean (standard deviation) unless otherwise stated. 25OHD, 25-hydroxyvitamin D.

b *P*-value from Kruskal-Wallis or ANOVA test for continuous variables and Chi-square test for categorical variables, as appropriate.

c Sample sizes differed for the following variables: asset index (*n* = 120 in group B; *n* = 113 in group D), 25OHD at enrollment (*n* = 198 in group A; *n* = 120 in group B; *n* = 132 in group C; *n* = 113 in group D; *n* = 121 in group E), secondary hyperparathyroidism (*n* = 198 in group A; *n* = 120 in group B; *n* = 132 in group C; *n* = 113 in group D; *n* = 121 in group E), 25OHD at delivery (*n* = 130 in group A; *n* = 118 in group B; *n* = 128 in group C; *n* = 120 in group E), 25OHD at 6 months postpartum (*n* = 114 in group A; *n* = *118* in group B; *n* = 128 in group C; *n* = 111 in group D; *n* = 119 in group E), and gestational duration (*n* = 195 in group A).

d Enrollment weight and height for BMI and samples for measurement of 25OHD were collected in the second trimester (17–24 weeks of gestation).

e Secondary hyperparathyroidism was defined 25OHD concentration below 30 nmol/L and iPTH concentration >6.82 pmol/L.

f Duration of exclusive breastfeeding was derived as the number of continuous weeks from birth to 26 weeks; however, all infants were assumed to be exclusively breastfed (received breastmilk only) in the first week of life.

**Table 2:** Concentrations of maternal bone-related biomarkers in the second trimester (17–24 weeks of gestation), at delivery, and at 6 months postpartum, by intervention group, in a pregnancy cohort in Dhaka, Bangladesh.

	Intervention group (prenatal;postpartum vitamin D dose, IU/week)									
	*N*	Placebo (0;0)	*N*	B (4200;0)	*N*	C (16,800;0)	*N*	D (28,000;0)	*N*	E (28,000;28,000)
		Geometric mean (95 % CI)		Geometric mean (95 % CI)		Geometric mean (95 % CI)		Geometric mean (95 % CI)		Geometric mean (95 % CI)
OPG (pg/mL)										
Enrolment	119	283.0 (260.7, 307.3)	112	285.5 (264.0, 308.8)	124	300.9 (278.3, 325.3)	114	289.1 (262.9, 317.9)	121	291.1 (269.2, 314.7)
Delivery	111	427.4 (384.5, 475.1)	111	427.3 (392.5, 465.1)	123	407.5 (362.2, 458.5)	106	409.4 (370.7, 452.1)	113	456.2 (416.1, 500.2)
6 mo. postpartum	112	266.3 (240.4, 294.9)	–	–	–	–	111	275.4 (258.1, 293.8)	117	267.2 (244.9, 291.6)
RANKL (pg/mL)										
Enrolment	120	13.1 (10.7, 16.2)	112	12.2 (9.6, 15.5)	125	11.5 (9.6, 13.8)	114	11.9 (9.5, 14.9)	121	12.0 (9.9, 14.5)
Delivery	112	13.4 (10.7, 16.7)	111	16.3 (13.3, 20.0)	124	13.0 (10.7, 15.7)	106	12.0 (9.8, 14.8)	113	15.7 (13.1, 18.8)
6 mo. postpartum	113	34.3 (29.7, 39.6)	–	–	–	–	111	34.1 (29.2, 39.7)	117	34.5 (30.4, 39.1)
OPG/RANKL (pg/mL/pg/mL)										
Enrolment	119	21.7 (17.2, 27.4)	112	23.3 (18.1, 30.0)	124	26.3 (21.2, 32.5)	114	24.3 (18.9, 31.3)	121	24.3 (19.6, 30.2)
Delivery	111	32.4 (25.1, 41.9)	111	26.2 (20.8, 32.9)	123	31.2 (24.8, 39.2)	106	34.0 (27.1, 42.7)	113	29.1 (23.7, 35.8)
6 mo. postpartum	112	7.7 (6.3, 9.5)	–	–	–	–	111	8.1 (6.8, 9.6)	117	7.8 (6.6, 9.1)
P1NP (μg/L)^a^										
Delivery	113	95.56 (85.26, 107.10)	111	96.69 (85.82, 108.94)	121	101.28 (91.20, 112.46)	106	90.15 (79.74, 101.92)	114	86.25 (75.48, 98.55)
6 mo. postpartum	113	31.97 (23.59, 43.33)	–	–	–	–	110	31.33 (23.24, 42.24)	118	40.70 (30.76, 53.86)
OC (μg/L)										
Enrolment	119	11.01 (10.08, 12.02)	112	10.94 (9.99, 11.98)	124	11.64 (10.67, 12.71)	114	10.68 (9.96, 11.46)	121	10.63 (9.75, 11.60)
Delivery	111	16.55 (14.78, 18.53)	111	15.90 (14.23, 17.77)	123	16.32 (14.59, 18.26)	106	14.82 (13.42, 16.37)	113	15.76 (14.26, 17.42)
6 mo. postpartum	112	25.49 (23.74, 27.38)	–	–	–	–	111	24.98 (23.35, 26.72)	117	25.04 (22.28, 28.13)
FGF23 (RU/mL)										
Enrolment	200	98.9 (87.1, 112.3)	120	103.0 (87.6, 121.1)	133	111.4 (95.3, 130.3)	114	89.9 (74.6, 108.3)	122	96.9 (82.0, 114.5)
Delivery	191	146.1 (128.0, 166.8)	111	133.8 (112.7, 159.0)	126	158.6 (135.7, 185.5)	106	131.8 (111.9, 155.3)	113	134.6 (115.4, 157.0)
6 mo. postpartum	115	82.3 (75.2, 90.1)					111	88.4 (81.1, 96.3)	119	88.5 (81.0, 96.6)
CTx (ng/mL)										
Enrolment	117	0.34 (0.29, 0.39)	112	0.33 (0.28, 0.37)	122	0.30 (0.27, 0.35)	106	0.33 (0.28, 0.38)	117	0.32 (0.28, 0.37)
Delivery^b^	114	0.56 (0.50, 0.63)	–	–	–	–	–	–	112	0.41 (0.36, 0.46)
6 mo. postpartum^b^	109	0.43 (0.38, 0.50)	–	–	–	–	–	–	108	0.46 (0.41, 0.52)

a Enrolment P1NP was not quantified.

b CTx at delivery in groups B, C and D, and postpartum CTx in group D, were measured but excluded from primary analyses (see Supplemental Methods for explanation).

### 3.2 Effect of vitamin D supplementation on bone biomarkers at delivery

In primary analyses, OPG, OPG/RANKL, P1NP, OC, RANKL, and FGF23 concentrations at delivery did not differ between any vitamin D group (B, C, or D + E) and placebo (Table 3; Supplemental Fig. 4). Similar results were observed in per-protocol analyses restricted to women who consumed ≥90 % of the assigned trial supplements throughout the prenatal period (Supplemental Table 3). Inferences were unchanged upon removal of outliers from the OPG and P1NP distribution (Supplemental Fig. 5). Mean CTx concentrations in group E (28,000;28,000 IU/week) were 27 % (95 % CI: −38, −13, *P* ≤ 0.001) lower than in the placebo group in both primary (Table 3, Supplemental Fig. 4) and per-protocol analyses (Supplemental Table 3). In post-hoc analyses including a correction factor for laboratory batch variation (affecting groups B, C, and D), CTx concentrations at delivery were significantly lower for each vitamin D intervention group compared to placebo, with the lowest CTx concentration in the two high-dose groups (Supplemental Table 4).

**Table 3:** Differences in bone biomarker concentrations between each vitamin D group and placebo group among pregnant women at delivery.

	Intervention group (prenatal vitamin D dose, IU/week)^a^						
	*N*	B (4200)	*P*^b^	C (16800)	*P*^b^	D + E (28000)	*P*^b^
		Percent difference (95 % CI)		Percent difference (95 % CI)		Percent difference (95 % CI)	
OPG	564	−0.46 (−12,12)	0.938	−8.6 (−19, 2.5)	0.123	−0.26 (−10,10)	0.961
OPG/RANKL	564	−20 (−42, 9.7)	0.164	−7.5 (−32, 26)	0.625	−5.0 (−28, 25)	0.718
P1NP	565	1.2 (−14, 20)	0.89	6.0 (−10, 25)	0.486	−7.8 (−20, 6.6)	0.271
OC	562	−1.6 (−12,11)	0.787	−4.3 (−15, 7.2)	0.449	−4.0 (−13, 6.2)	0.430
RANKL^c^	566	32 (−6.2, 86)	0.111	2.9 (−26, 44)	0.867	9.9 (−18, 48)	0.535
FGF23^c^	647	−9.1 (−26, 11)	0.356	6.2 (−13, 29)	0.544	−7.0 (−21,10)	0.400
CTx^d^	226	–	–	–	–	−27 (−38, −13)	<0.001

a Groups D and E are combined in these analyses since they were identical in prenatal dose with the exception of CTx, which is presented for group E only.

b *P*-value of mean difference in each intervention group versus placebo, adjusting for enrollment (second trimester) biomarker concentration (except P1NP, due to unavailability of enrollment P1NP data).

c Tobit regression to account for left censoring of RANKL distributions and right censoring of FGF23 distribution.

d The effect of vitamin D on CTx concentration is shown for group E versus group A; see supplementary methods for explanation.

### 3.3 Effect of vitamin D supplementation on bone biomarkers at 6 months postpartum

In primary analyses, OPG, OPG/RANKL, P1NP, OC, RANKL, and FGF23 concentrations did not differ between either group D or E and placebo at 6 months postpartum (Table 4; Supplemental Fig. 6). In per-protocol analyses limited to women with ≥90 % adherence, FGF23 concentrations in group D were statistically greater than placebo, but inferences were unchanged from primary analyses for all other biomarkers (Supplemental Table 5). Inferences were also similar to primary analyses upon removal of aforementioned outliers, such that there was no difference in bone biomarker concentrations between the vitamin D intervention groups relative to placebo (Supplemental Fig. 5).

**Table 4:** Differences in bone biomarker concentrations between vitamin D groups D and E compared to placebo among women at 6 months postpartum.

	Intervention group (prenatal;postpartum vitamin D dose, IU/week)				
	*N*	D (28,000;0)	*P*^a^	E (28,000;28,000)	*P*^a^
		Percent difference (95 % CI)		Percent difference (95 % CI)	
OPG	340	3.2 (−8.5, 17)	0.603	−0.24 (−11,12)	0.968
OPG/RANKL	340	3.9 (−19, 34)	0.765	−0.98 (−23, 27)	0.938
P1NP^b^	341	1.5 (−45, 86)	0.961	46 (−19, 164)	0.207
OC	340	−1.2 (−12, 11)	0.843	−0.84 (−12, 12)	0.888
RANKL^b^	341	−0.32 (−19, 22)	0.975	1.4 (−17, 24)	0.890
FGF23	345	9.5 (−2.7, 23)	0.131	8.2 (−3.6, 21)	0.183
CTx^c^	217	–	–	7.1 (−9.6, 27)	0.426

a *P*-value of mean difference each intervention group versus placebo, adjusting for enrollment (second trimester) biomarker concentration (except P1NP, due to unavailability of enrollment P1NP data).

b Based on tobit regression to account for left censoring of P1NP and RANKL distributions.

c The effect of vitamin D on CTx concentration is shown for group E versus group A only; see Supplemental Methods for explanation.

CTx concentrations in group E did not differ from placebo at 6 months postpartum in primary (Table 4; Supplemental Fig. 6) or per-protocol analyses (Supplemental Table 5). Compared to the primary analysis, the magnitude of the percent difference in group E relative to placebo increased upon adjustment for delivery CTx concentrations (rather than enrollment CTx) in a post-hoc analysis; however, confidence intervals remained wide and inferences were unchanged (mean difference = 14 %; 95 % CI: −5.3, 37; *P* = 0.168). Postpartum CTx concentrations did not differ significantly between group D and placebo upon correction for laboratory batch variation (Supplemental Table 4). From enrollment to delivery, mean CTx concentrations increased in both the placebo group and group E; however, delivery CTx concentrations in group E were significantly lower than the placebo group, such that the magnitude of increase appeared greater in the placebo group (Supplemental Fig. 7). CTx concentrations continued to increase in group E after delivery but decreased in the placebo group such that they were similar at 6 months postpartum (Supplemental Fig. 7).

### 3.4 iPTH as a mediator of the effect of vitamin D supplementation on CTx at delivery

**Fig. 2:**
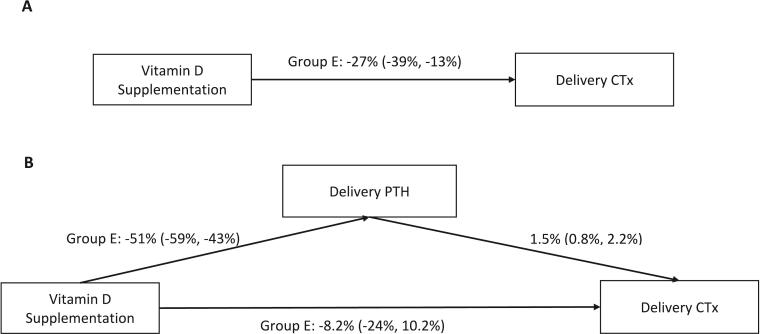
The total effects (A) and direct and indirect effects (B) of prenatal vitamin D supplementation on CTx concentrations at delivery, mediated by parathyroid hormone (PTH) concentrations at delivery. Effect estimates represent the percent difference (95 % confidence interval) in CTx concentrations in group E (28,000;28,000 IU/week, prenatal;postpartum) compared to group A (placebo), the reference in all models, for each 10 % increase in PTH. The proportion of the total effect of vitamin D on CTx when iPTH was accounted for in the model was calculated as follows −0.082/−0.27 ∗ 100.

Mediation analyses demonstrated a statistically significant direct effect of high-dose prenatal vitamin D supplementation (28,000 IU/week) on iPTH concentrations at delivery, which partly mediated the effect of vitamin D on CTx (Fig. 2). Specifically, there was a positive association between CTx and iPTH at delivery, such that CTx increased by 1.5 % (95 % CI: 0.8, 2.2; *P* < 0.001) for every 10 % increase in iPTH. Most of the effect of vitamin D supplementation on CTx was mediated by iPTH; the direct effect of vitamin D on CTx accounted for 30 % of the total effect following adjustment for iPTH in the final model (Fig. 2). Similarly, post-hoc mediation analysis using 25OHD at delivery as the exposure variable demonstrated the same positive association between CTx and iPTH at delivery and significant mediation of the association between 25OHD and CTx by iPTH (Supplemental Fig. 8).

### 3.5 Timing of CTx sample collection

There were no significant differences in the time of day of CTx sample collection between intervention groups (Supplemental Fig. 9).

## 4 Discussion

In a placebo-controlled dose-ranging trial, maternal vitamin D supplementation had minimal effects on markers of maternal bone remodeling from mid-pregnancy to the first 6 months postpartum. While high-dose supplementation markedly suppressed plasma CTx concentrations by delivery, reflecting a decrease in bone resorption with improvement of maternal vitamin D status, this effect was not sustained by continued vitamin D supplementation during the postpartum period. Effects on RANKL were not observed either prenatally or postpartum, nor did maternal vitamin D supplementation markedly influence bone formation and metabolism, as measured by OPG, OC, P1NP and FGF23. Therefore, the present findings support the hypothesis that high-dose vitamin D supplementation reduces bone resorption during pregnancy, however these effects are not sustained by 6 months postpartum in a population with habitually low levels of 25OHD prior to intervention.

The present finding of lower CTx concentrations following prenatal vitamin D supplementation are consistent with findings from the Maternal Vitamin D Osteoporosis Study (MAVIDOS), which demonstrated a lower mean increase in urinary CTx in late gestation following intervention with a relatively modest prenatal vitamin D dose (1000 IU/d) (Curtis et al., 2021). Although enrollment to the MAVIDOS trial was restricted to women with 25OHD concentrations ≥25 nmol/L, the authors describe a “threshold effect” whereby the effect of the intervention was pronounced among women with baseline concentrations below 50 nmol/L, a conventional cut-off for vitamin D insufficiency; among women with low vitamin D status, the conditional rise in CTx was greater among women who received placebo relative to the vitamin D intervention (Δ CTx z-score: 0.28 vs −0.15; *P* < 0.01), whereas the change was similar between groups among women with baseline 25OHD concentrations ≥50 nmol/L (Curtis et al., 2021). Our findings, together with that of the MAVIDOS trial, support earlier evidence from an observational study of an inverse association between circulating 25OHD and CTx concentrations in the second and third trimesters (Haliloglu et al., 2011), and suggest the effect of supplementation may be greatest among women who enter pregnancy with a low vitamin D status. Nonetheless, the magnitude of the effect size (27 %) we observed is lower than that reported from pharmacological trials aimed at attenuating postmenopausal-related osteoporosis (Szulc et al., 2017), and hence the clinical significance of our findings should be interpreted alongside the null effects on other biomarkers of bone formation and resorption.

The reason for the lack of an effect of vitamin D supplementation on other biomarkers of bone turnover is unclear. We expected to find a comparable decline in RANKL as was observed for CTx because both biomarkers reflect bone resorption (Wheater et al., 2013); however, concentrations of RANKL remained similar between the vitamin D intervention groups and placebo at delivery and 6 months postpartum. Effects on OPG were also not observed, and the OPG/RANKL ratio was unchanged following intervention. Although data from human trials is limited, recent evidence from non-pregnant adults in Norway similarly showed no effect of weekly high-dose vitamin D supplementation on OPG and RANKL in a population with a relatively low vitamin D status (Jorde et al., 2019). Furthermore, a recent secondary analysis of a dose-ranging UK-based trial found no effect of vitamin D supplementation (12,000, 24,000 and 48,000 IU/month) on OPG and RANKL concentrations in participants 70 years of age, nor were any effects observed when analyses were restricted to participants with baseline 25OHD concentrations <25 nmol/L (Christodoulou et al., 2022). In extension of these findings, the null effects observed in our study support the findings of Wei and colleagues who reported no between-group differences for changes in BMD of the spine and femoral neck between 12-20 weeks of gestation and 0–14 weeks postpartum across three doses of supplemental vitamin D (400, 2000 and 4000 IU/d) in a randomized trial in the US (Wei et al., 2017).

As reviewed by van Ballejooigen, 1,25(OH)2D3 stimulates osteoblast expression of OC through a vitamin K-dependent pathway, such that both vitamins work in tandem to promote carboxylation of OC (van Ballegooijen et al., 2017). Given the randomized design, we assumed similar distributions of dietary vitamin K intake and circulating vitamin K vitamers between intervention groups. Vitamin K deficiency is uncommon (World Health Organization and Food and Agriculture Organization of the United Nations, 2004), yet it is possible that a low vitamin K status in the present cohort, through poor dietary intake or malabsorption, precluded any synergistic benefit of vitamin D supplementation on vitamin K-dependent protein post-translational modification (van Ballegooijen et al., 2017). The reason for the null effects on P1NP is also unclear; results from two studies have yielded inconsistent findings for an effect of vitamin D on P1NP concentrations, albeit in non-pregnant adults (Jorde et al., 2019; Christodoulou et al., 2022).

Evidence for an effect of vitamin D supplementation on FGF23 concentrations is mixed and complicated by variability in assay selection since different peptides may be targeted. While a recent meta-analysis found that vitamin D supplementation increases serum intact-FGF23 among non-pregnant adults with low vitamin D status (<50 nmol/L), the pooled analysis for effects on c-terminal FGF23 was limited to only two trials for which the effects were not statistically significant (Charoenngam et al., 2019). In a restricted analysis including only participants who were highly compliant with the intervention, we found greater c-terminal FGF23 concentrations in group D relative to placebo at 6 months postpartum. However, we remain cautious in our interpretation of these findings given their inconsistency with primary analyses, relatively large confidence intervals surrounding the effect estimates, and lack of an intervention effect among participants who continued supplementation throughout the postpartum period.

In a previous report from the MDIG trial, prenatal vitamin D supplementation was shown to suppress iPTH secretion in a dose-dependent manner (Roth et al., 2018). Here, we present new evidence that iPTH mediated the effect of vitamin D supplementation on CTx, in agreement with previous literature suggesting the protective effective of vitamin D on bone metabolism is due in part to the reduction in circulating PTH (Jorde et al., 2019; Pasco et al., 2004; Gao et al., 2017). In response to declining serum calcium, the rise in PTH stimulates bone resorption by indirectly promoting osteoclast differentiation and increasing collagenase activity in osteoblasts (McSheehy and Chambers, 1986; Zhao et al., 1999). Collagenase activity stimulates the attachment of osteoclasts to the collagen surface of bone, where resorptive activity by osteoclasts then occurs (Everts et al., 2022). CTx is a collagen by-product of bone related resorption, and a specific degradation product of the osteoclast secreted protease cathepsin K (Wheater et al., 2013), which is responsible for releasing collagen derived bone fragments into the circulation (Garnero et al., 2003). The greater CTx concentrations of the placebo group in this study therefore likely represents a cascade of maternal bone resorption that is prompted by a rise in maternal PTH, a process which we have shown to be at least partially suppressed by high-dose prenatal vitamin D supplementation.

However, postpartum continuation of high-dose vitamin D supplementation resulted in similar CTx concentrations as the placebo group by 6 months postpartum. Owing to batch-to-batch variation in CTx, we did not obtain robust measures of postpartum CTx in the group randomized to receive the same intervention dose in the prenatal period only (28,000;0 IU/week); we therefore could not definitively isolate the prenatal effect (i.e., group D versus group E). A post-hoc analysis revealed a small and non-significant postpartum increase in CTx concentrations in women who continued to receive vitamin D supplementation. It is possible that compensatory mechanisms in the postpartum period overcome the effects of PTH-mediated bone loss due to vitamin D deficiency during pregnancy.

We acknowledge several limitations of the present study. First, this was a secondary analysis among a sub-set of original trial participants which was not planned in tandem with the original trial. Second, given this was a pregnant population, blood samples were collected in a non-fasting state. CTx has been shown to demonstrate circadian variation (Qvist et al., 2002), hence, fasting samples are recommended to limit intra-individual variation in the clinical setting (Qvist et al., 2002). Although the distribution of time of day of sample collection was similar across intervention groups, and the extent of intra-individual diurnal variation was expected to be similar across trial arms, excessive within-group variation in CTx could have biased estimates of between-group differences towards the null. We also acknowledge the laboratory overestimation of CTx concentrations that affected some batches, such that our primary analysis was limited to participants in group E and placebo only. Third, the low dietary calcium intakes that are common in Bangladesh may exacerbate bone resorption during pregnancy and postpartum (Cullers et al., 2019), but the calcium supplementation (500 mg/d) provided to all women throughout the intervention period may have attenuated the observed effects of the vitamin D intervention (Roth et al., 2018; Roth et al., 2015). Lastly, while bone turnover markers have been accepted as a useful tool for the early assessment of response to treatment for osteoporosis when compared with conventional dual-energy x-ray absorptiometry (DXA) or alternative imaging scans (Wheater et al., 2013; Bergmann et al., 2009), we lacked direct measurements of bone mass and maternal specimens were only available up to 6 months postpartum, such that we did not measure longer-term effects on bone metabolism and recovery post-lactation.

### 4.1 Conclusion

In a population with a high prevalence of vitamin D deficiency, prenatal vitamin D supplementation from the second trimester suppressed circulating CTx by delivery, an effect that was partly mediated by the suppression of circulating iPTH; however, this effect was not sustained by continued vitamin D supplementation to 6 months postpartum. Effects of vitamin D on other biomarkers of bone turnover, specifically OPG, OPG/RANKL, OC, P1NP, RANKL and FGF23 were not observed. These findings do not strongly suggest an overall beneficial effect of high-dose vitamin D supplementation on bone mineral metabolism during pregnancy and the postpartum period, even among women who are vitamin D deficient during pregnancy. Therefore, further evidence is needed to substantiate potential longer-term benefits of vitamin D on maternal bone health during the postpartum period.

## CRediT authorship contribution statement

**Christine Krupa:** Formal analysis, Visualization, Writing – original draft. **Huma Qamar:** Formal analysis, Visualization, Writing – review & editing. **Karen M. O'Callaghan:** Methodology, Supervision, Writing – review & editing. **Akpevwe Onoyovwi:** Investigation, Writing – review & editing. **Abdullah Al Mahmud:** Supervision, Writing – review & editing. **Tahmeed Ahmed:** Writing – review & editing. **Alison D. Gernand:** Writing – review & editing. **Daniel E. Roth:** Conceptualization, Methodology, Supervision, Writing – review & editing.

## Declaration of competing interest

The authors have nothing to disclose.

## Data Availability

Data described in the manuscript, code book, and analytic code will be made available upon reasonable request to the authors. De-identified individual participant data will be provided for use in secondary data analyses approved by an independent research ethics board, and data requestors will be required to sign a data access agreement.

## Appendix A Supplementary data

Supplementary material Image 1
