# Supplementary material for: Prenatal but not continued postpartum vitamin D supplementation reduces maternal bone resorption as measured by C-terminal telopeptide of type 1 collagen without effects on other biomarkers of bone metabolism
Source: Endocr Metab Sci. 2024 Mar 31;14:None. doi: 10.1016/j.endmts.2023.100154 (PMC10914667; doi:10.1016/j.endmts.2023.100154)
Supplement: Supplementary file 1 — Supplementary material [file mmc1.pdf]

Supplementary Appendix

Prenatal but not continued postpartum vitamin D reduces maternal bone resorption as measured by C-terminal telopeptide of type 1 collagen without effects on other biomarkers of bone metabolism

Christine Krupa[1], B.Sc., Huma Qamar[1], M.Sc., Karen M. O'Callaghan[1,2], Ph.D., Akpevwe Onoyovwi[1], M.Sc., Abdullah Al Mahmud[3], M.S.S., M.M.Sc., Tahmeed Ahmed[3] M.B., B.S., Alison D. Gernand[4], Ph.D., Daniel E. Roth[1,5,6], M.D., Ph.D.

[1]Centre for Global Child Health, Hospital for Sick Children, Toronto, Canada

[2]Department of Nutritional Sciences, King's College London, United Kingdom

[3]Nutrition and Clinical Services Division, International Centre for Diarrhoeal Disease Research, Dhaka, Bangladesh

[4]Department of Nutritional Sciences, The Pennsylvania State University, Pennsylvania, United States

[5]Department of Nutritional Sciences, University of Toronto, Toronto, Canada

[6]Department of Paediatrics, Hospital for Sick Children and University of Toronto, Toronto, Canada

Address correspondence to:
Daniel E. Roth
Department of Paediatrics
The Hospital for Sick Children and University of Toronto
686 Bay Street, Toronto, Canada
daniel.roth@sickkids.ca

Table of Contents

Supplemental Methods 2

Laboratory Analyses *2*

Sensitivity Analyses to account for laboratory batch variation in CTx concentration *2*

Supplemental Table 1: Assay information and performance indicators for bone-related biomarker measurements.......... 3

Supplemental Table 2: Maternal characteristics of study sample and sample ineligible for the maternal bone biomarker study. 4

Supplemental Table 3: Percent differences between the average concentrations of biomarkers of bone metabolism in each vitamin D treatment group versus the placebo group at delivery following a per-protocol approach. 5

Supplemental Table 4: Effect of vitamin D supplementation on CTx concentrations at delivery and at 6 months postpartum after applying a correction factor to account for overestimation of CTx concentrations in groups B, C, and D 6

Supplemental Table 5: Percent differences between the average concentrations of biomarkers of bone metabolism in each vitamin D treatment group versus placebo at 6 months postpartum following a per-protocol approach. 7

Supplemental Figure 1: Per-protocol participant selection flow diagram. 8

Supplemental Figure 2: Flow diagram for CTx samples, by laboratory batch and vitamin D intervention group 9

Supplemental Figure 3: Batch-related variation in CTx assay performance. CTx was measured in-house using a commercially-available ELISA kit. 10

Supplemental Figure 4: Bone biomarker geometric mean, and 95% CI by vitamin D intervention group at delivery. 11

Supplemental Figure 5: Box plots showing the distribution of maternal bone biomarkers included in sensitivity analyses in which statistical outliers were removed. 12

Supplemental Figure 6: Bone biomarker geometric mean, and 95% CI by vitamin D intervention group at 6 months postpartum.......... 13

Supplemental Figure 7: Geometric mean CTx concentrations (ng/mL) of women who contributed biomarker data at enrolment (17-24 weeks' gestation), at delivery, and at 6 months postpartum, by vitamin D intervention group (*N*=209).......... 14

Supplemental Figure 8: The total effects (A) and direct and indirect effects (B) of maternal delivery 25-hydroxyvitamin D (25OHD) on CTx concentrations at delivery, mediated by parathyroid hormone (PTH) concentrations at delivery.......... 15

Supplemental Figure 9: Time of day of CTx sample collection, stratified by vitamin D treatment group 16

Supplemental Methods

Laboratory Analyses

Batched analysis of CTx was conducted as follows: batch 1 included delivery and postpartum samples from groups A and E; batch 2 included delivery samples from groups B, C, and D and postpartum samples from group D; batch 3 included enrollment samples from all groups (**Supplemental Figure 2**). Batch 3 was incorporated based on a post-hoc decision to examine potential between-group differences in enrolment CTx concentrations. Groups D and E were both randomized to receive a prenatal dose of 28000 IU vitamin D/week and were therefore expected to have similar distributions of biomarker concentrations at delivery; however, we observed higher average delivery CTx concentrations in group D versus group E, which we attributed to a systematic over-estimation of CTx in batch 2 (**Supplemental Figure 3**). Repeat testing of all batch 2 samples was unfeasible; therefore, we made a post-hoc decision to limit primary analyses of effects on CTx to the comparison of the high-dose prenatal and postpartum intervention in group E versus placebo (i.e., corresponding to laboratory analyses performed in batch 1 and 3). CTx concentrations of groups B, C, and D at delivery and group D at 6-months postpartum (batch 2 data) were included in sensitivity analyses using a correction factor to account for batch-to-batch variation.

Sensitivity Analyses to account for laboratory batch variation in CTx concentration

In post-hoc analyses, we applied a correction factor to delivery CTx concentrations in groups B, C and D and 6-month postpartum CTx concentrations in group D to account for the overestimation of CTx in batch 2 (Supplementary Figure 3). The correction factor was determined based on the assumption that the distribution and mean CTx concentrations at delivery should be similar between groups D and groups E due to randomization to an identical

prenatal vitamin D dosing regimen (28000 IU/week) and similar CTx concentrations at enrollment across treatment groups. Therefore, the correction factor was calculated as the mean ln(CTx) difference between groups E (mean: -0.894 ln[ng/mL]) and D (mean: -0.635 ln[ng/mL]) at delivery (correction factor: -0.260 ln[ng/mL]); this correction factor was applied to all natural log-transformed CTx concentrations in groups B, C, and D at delivery and in group D at 6 months postpartum, whereby corrected ln(CTx [ng/mL]) = ln(uncorrected value [ng/mL] – 0.260 [ng/mL]). Using corrected CTx concentrations as the dependent variable, linear regression models were constructed to assess the effect of the vitamin D intervention relative to placebo and presented as mean percent differences with 95% CI.

Supplemental Table 1: Assay information and performance indicators for bone-related biomarker measurements.

Biochemical measure	Biological matrix	Laboratory (institution)	Method	Assay kit/platform (manufacturer)	LLOQ	ULOQ	Imputed value if <LLOQ[a]	Imputated value if >ULOQ[a]	Samples <LLOQ *n* (%)	Samples >ULOQ *n* (%)	Inter-assay CV	Intra-assay CV
iPTH	Plasma	AFBM (SickKids)	ELISA	Kit #60-3100 (Immutopics, California)	1.58 pmol/L	-	0.79	NR	Baseline: 33 (4.5)<br>Delivery: 87 (14)<br>6 mo PP: 19 (3.2)	N/A	16%	4.2%
25(OH)D[b]	Serum	AFBM (SickKids)	LC-MS/MS	Agilent 1290 HPLC system (Agilent Technologies; Q-TRAP 5500 mass spectrometer (Sciex)	1.25 nmol/L	-	NR	NR	N/A	N/A	10%	5.0%
FGF23	Plasma	AFBM (SickKids)	ELISA	Kit #60-600, (Immutopics, California)	9.1 RU/mL	1415 RU/mL	4.55 RU/mL	1415 RU/mL	Baseline: 2 (0.27)<br>Delivery: 0<br>6 mo PP: 0	Baseline: 13 (1.8)<br>Delivery: 18 (3.0)<br>6 mo PP: 0	6.0%	3.0%
OPG	Plasma	AFBM (SickKids)	Luminex/ Millipore	HBNMAG-51-K Luminex/Millipore magnetic beads kits (Millipore, Massachusetts)	7.32 pg/mL	-	3.66 pg/mL	NR	Baseline: 0<br>Delivery: 1 (0.17)<br>6 mo PP: 1 (0.29)	N/A	7.1%	4.8%
OC	Plasma	AFBM (SickKids)	Luminex/ Millipore	HBNMAG-51-K Luminex/Millipore magnetic beads kits (Millipore, Massachusetts)	0.146 µg/L	600 µg/L	0.073 µg/L	600 µg/L	Baseline: 1 (0.12)<br>Delivery: 0<br>6 mo PP: 1 (0.29)	Baseline: 0<br>Delivery: 2 (0.34)<br>6 mo PP: 0	12%	4.3%
RANKL	Plasma	AFBM (SickKids)	Luminex/ Millipore	Kit HRNKLMAG-5QK-01 (MyBioSource, California)	4.88 pg/mL	-	2.44 pg/mL	NR	Baseline: 96 (16)<br>Delivery: 75 (13)<br>6 mo PP: 7 (2.0)	N/A	6.6%	5.3%
CTx	Plasma	SickKids	ELISA	Kit AC-02F1 (Immunodiagnostic Systems, United Kingdom)	V1:0.159 ng/mL<br>V2: 0.174 ng/mL<br>V3: 0.149 ng/mL	-	V1:0.0795 ng/mL<br>V2: 0.087 ng/mL<br>V3: 0.0745 ng/mL	NR	Baseline: 17 (2.9)<br>Delivery: 0<br>6 mo PP: 0	Baseline: 0<br>Delivery: 3 (0.52)<br>6 mo PP: 0	3.8%	5.3%
P1NP[c]	Serum	SickKids	ELISA	Kit MBS2504819 (MyBioSource, California)	7.815 µg/L	-	3.9075 µg/L	NR	Baseline: N/A<br>Delivery: 5 (0.88)<br>6 mo PP: 109 (32)	N/A	18%	10%

25(OH)D, 25-hydroxyvitamin D; 6 mo PP, 6 months postpartum; AFBM, Analytical Facility for Bioactive Molecules; CTx, carboxy terminal telopeptide of type 1 collagen; ELISA, enzyme-linked immunosorbent assay; FGF23, fibroblast growth factor 23; iPTH, intact parathyroid hormone; LC-MS/MS, liquid. chromatography-tandem mass spectrometry; LLOQ; lower limit of quantification; N/A, not applicable; NR, not required; OC, osteocalcin; OPG, osteoprotegerin; P1NP, procollagen type 1 N-terminal propeptide; RANKL, receptor activator nuclear factor kappa-B ligand; SickKids, Hospital for Sick Children, Toronto, Canada; V1, kit version 1; V2, kit version 2; V3, kit version 3.

[a] NR denoted for biomarkers for which all measured concentrations were above the LLOQ or below the ULOQ.

[b] The AFBM lab participated in the Vitamin D External Quality Assessment Scheme (DEQAS). Only 25(OH)D3 concentrations were used in analyses because 25(OH)D2 was always undetectable in this cohort.

[c] Samples diluted 500-fold; samples went through 1 freeze-thaw cycle before analysis.

Supplemental Table 2: Maternal characteristics of study sample and sample ineligible for the maternal bone biomarker study.

Characteristic[a]	Eligible (n=690)	Ineligible[b] (n=608)	*P*[c]
Age (years)	22 (20, 26)	22 (20, 25)	0.482
Height (cm), *mean* ± *SD*	151.0 ± 5.6	150.9 ± 5.2	0.825
Asset Index[d]			0.414
1 (lowest)	137 (20)	124 (21)	
2	127 (18)	124 (21)	
3	132 (19)	124 (21)	
4	148 (22)	109 (18)	
5 (highest)	144 (21)	111 (19)	
Education, n (%)			0.143
No Education	26 (3.8)	32 (5.3)	
Primary incomplete	133 (19)	144 (24)	
Primary complete	97 (14)	82 (13)	
Secondary incomplete	270 (39)	228 (38)	
Secondary complete	164 (24)	122 (20)	
Occupation[4], n (%)			0.294
Unemployed	651 (94)	551 (93)	
Employed	39 (5.7)	42 (7.1)	
Serum 25(OH)D at enrollment in 2nd trimester (nmol/L) [4]	25.2 (17.0, 33.9)	26.0 (17.6, 35.7)	0.291
Gestational duration (weeks) [4]	39 (38, 40)	39 (38, 40)	0.321
Exclusive breastfeeding duration (weeks)	13 (5, 22)	13 (6, 20)	0.949

[a] Data presented as median (25th percentile, 75th percentile) unless otherwise stated.
[b] Participants in the MDIG trial who did not meet inclusion criteria for the maternal bone biomarker sub-study.
[c] P-value from t-tests or Kruskal-Wallis tests for continuous variables and Chi-square test for categorical variables.
[d] Sample size differed by variable: socioeconomic status (eligible *n*=688, ineligible *n*=592), occupation (eligible *n*=690, ineligible *n*=593), 25(OH)D (eligible *n*=684, ineligible *n*=607), gestational duration (eligible *n*=685, ineligible *n*=569).

Supplemental Table 3: Percent differences between the average concentrations of biomarkers of bone metabolism in each vitamin D treatment group versus the placebo group at delivery following a per-protocol approach.

		**Intervention Group** (prenatal vitamin D dose, IU/week)					
		**B** (4200)		**C** (16800)		**D+E** (28000)	
	N	**Percent Difference (95% CI)**	***P*[a]**	**Percent Difference (95% CI)**	***P*[a]**	**Percent Difference (95% CI)**	***P*[1]**
**OPG**	522	1.4 (-10, 15)	0.83	-10 (-20, 0.97)	0.07	-0.39 (-10, 11)	0.94
**OPG/RANKL**	522	-11 (-36, 25)	0.51	-2.4 (-29, 34)	0.88	1.2 (-24, 34)	0.94
**P1NP[b]**	526	-1.1 (-17, 17)	0.90	6.1 (-10, 25)	0.48	-7.8 (-20, 6.8)	0.28
**OC**	520	1.6 (-9.9, 15)	0.80	-6.1 (-16, 5.5)	0.29	-4.7 (-14, 5.7)	0.36
**RANKL[b]**	524	16 (-19, 65)	0.42	-6.3 (-33, 32)	0.71	2.6 (-24, 39)	0.87
**FGF23[b]**	601	-9.0 (-26, 12)	0.38	5.0 (-14, 28)	0.63	-7.3 (-22, 10)	0.39
**CTx[c]**	213	–	–	–	–	-27 (-38, -13)	0.001

[a] Per-protocol sensitivity analyses were limited to participants who consumed ≥90% of the assigned trial supplements. P-value of mean difference each treatment group versus placebo, adjusting for enrollment (second trimester) biomarker concentration (except P1NP, due to unavailability of enrollment P1NP data).

[b] Effect of vitamin D supplementation on biomarker concentration was ascertained using tobit regression to account for left censoring of P1NP and RANKL distributions and right censoring of FGF23 distribution. This was a result of a relatively large proportion of P1NP and RANKL concentrations below the lower limit of quantification and FGF23 concentration above the limits of quantification of the assay, respectively.

[c] The effect of vitamin D on CTx concentration is shown for group E versus group A only; see Supplementary Methods section for explanation.

Supplemental Table 4: Effect of vitamin D supplementation on CTx concentrations at delivery and at 6 months postpartum after applying a correction factor to account for overestimation of CTx concentrations in groups B, C, and D[a].

		**Intervention Group** (prenatal;postpartum vitamin D dose, IU/week)							
		**B** (4200;0)		**C** (16800;0)		**D** (28000;0)		**E** (28000;28000)	
	**N**	**% Mean Difference (95% CI)**	**P value[b]**	**% Mean Difference (95% CI)**	**P value[b]**	**% Mean Difference (95% CI)**	**P value[b]**	**% Mean Difference (95% CI)**	**P value[2]**
**Corrected delivery CTx**	559	-19 (-32, -4.5)	0.013	-21 (-33, -6.3)	0.006	-27 (-39, -13)	0.001	-26 (-38, -13)	<0.001
**Corrected postpartum CTx**	317	–	–	–	–	0.54 (-15, 19)	0.950	7.0 (-9.3, 26)	0.419

[a] The correction factor (-0.260 ln[ng/mL]) was applied to delivery and postpartum ln(CTX) concentrations of women randomized to groups B, C, and D; the correction factor corresponded to the mean difference in ln(CTX) concentrations between groups E and D.
[b] P value for mean difference in each intervention group versus placebo, adjusting for enrollment (second trimester) CTx concentration.

Supplemental Table 5: Percent differences between the average concentrations of biomarkers of bone metabolism in each vitamin D treatment group versus placebo at 6 months postpartum following a per-protocol approach.

		**Intervention Group** (prenatal;postpartum vitamin D dose, IU/week)			
		**D** (28000;0)		**E** (28000;28000)	
	N	**Percent Difference (95% CI)**	***P*[a]**	**Percent Difference (95% CI)**	***P*[a]**
**OPG**	272	-2.0 (-15, 13)	0.78	-2.2 (-15, 12)	0.76
**OPG/RANKL**	272	-8.6 (-31, 22)	0.54	-9.6 (-31, 19)	0.47
**P1NP[b]**	273	4.5 (-48, 110)	0.90	35 (-30, 162)	0.37
**OC**	272	-1.6 (-14, 13)	0.82	-0.53 (-13, 14)	0.94
**RANKL[b]**	272	7.9 (-14, 35)	0.50	8.3 (-13, 34)	0.47
**FGF23**	276	16 (2.1, 33)	0.024	11 (-2.0, 26)	0.10
**CTx[c]**	183	–	–	8.1 (-10, 30)	0.42

[a] Per-protocol sensitivity analyses were limited to participants who consumed ≥90% of the assigned trial supplements. P-value of mean difference in each intervention group versus placebo, adjusting for enrollment (second trimester) biomarker concentration (except P1NP, due to unavailability of enrollment P1NP data).
[b] Based on tobit regression to account for left censoring of P1NP and RANKL distributions.
[c] The effect of vitamin D on CTx concentration is shown for group E versus group A only; see Supplementary Methods section for explanation.

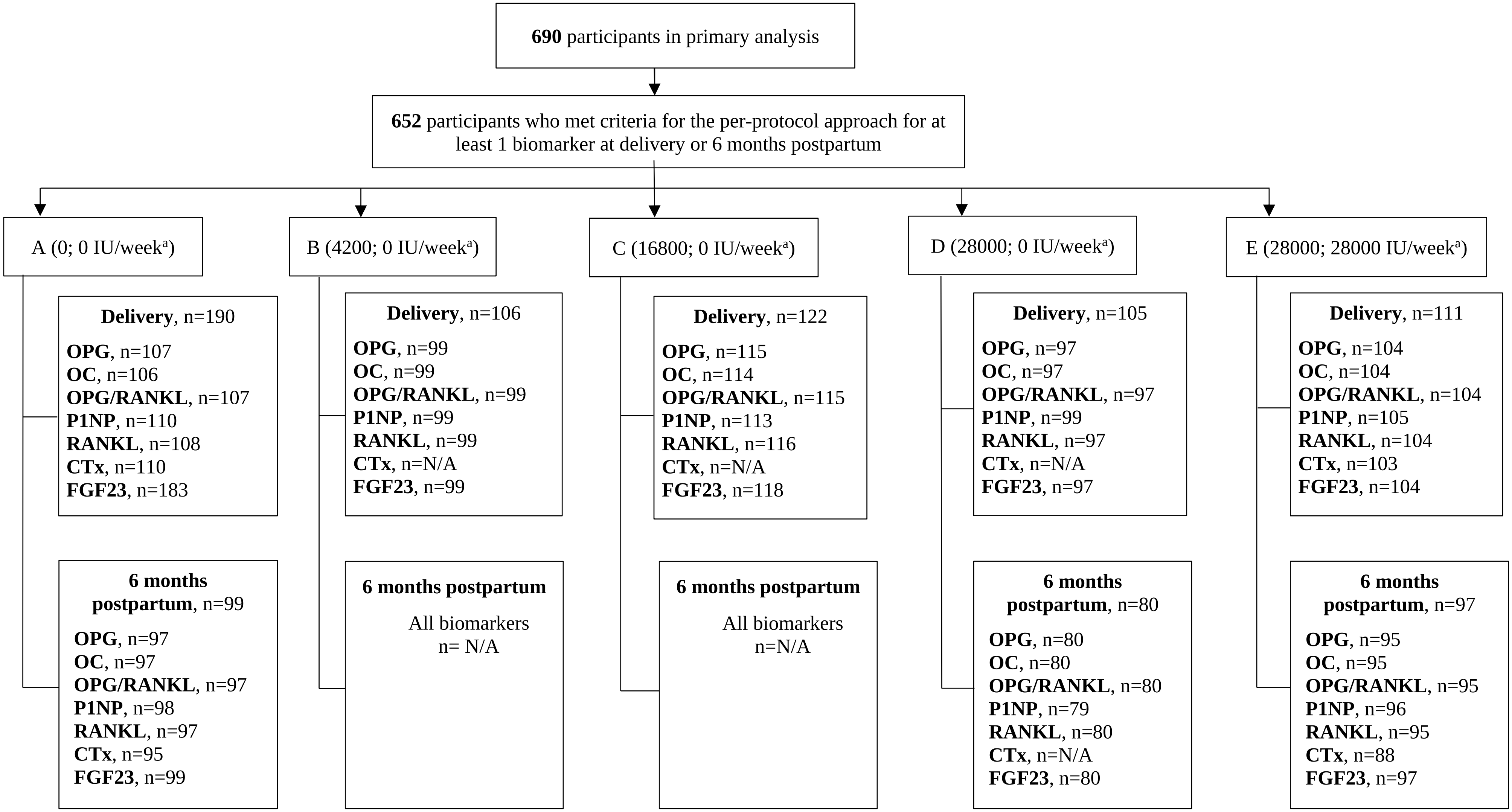

Supplemental Figure 1: Per-protocol participant selection flow diagram. Per-protocol analyses were restricted to women who consumed ≥90% of the assigned trial supplements and did not consume any non-study vitamin D/calcium supplements. In total, 652 participants met criteria for the per-protocol approach for delivery (n=634) and/or 6 months postpartum (n=276).

[a] Represents the prenatal; postpartum vitamin D intervention assigned at randomization.

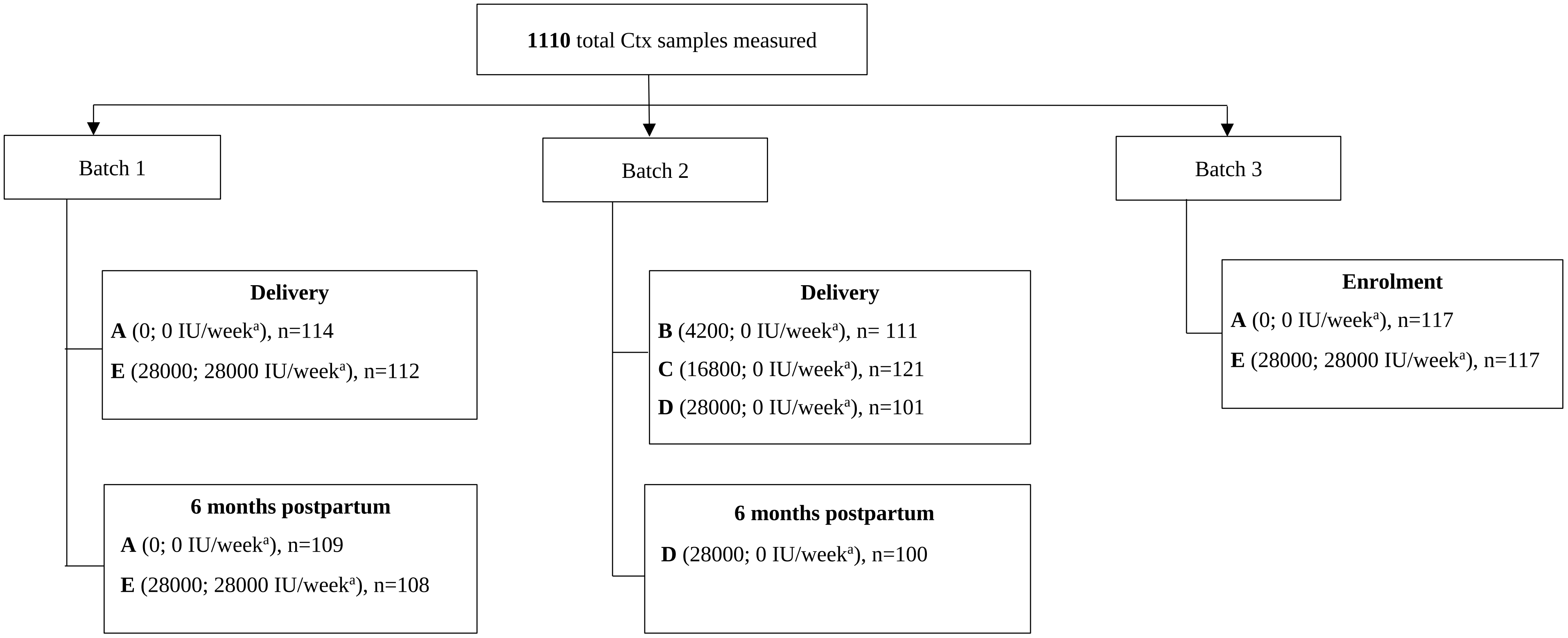

Supplemental Figure 2: Flow diagram for CTx samples, by laboratory batch and vitamin D intervention group. See Supplementary Methods section for further details.
[a] Represents the prenatal; postpartum vitamin D intervention assigned at randomization.

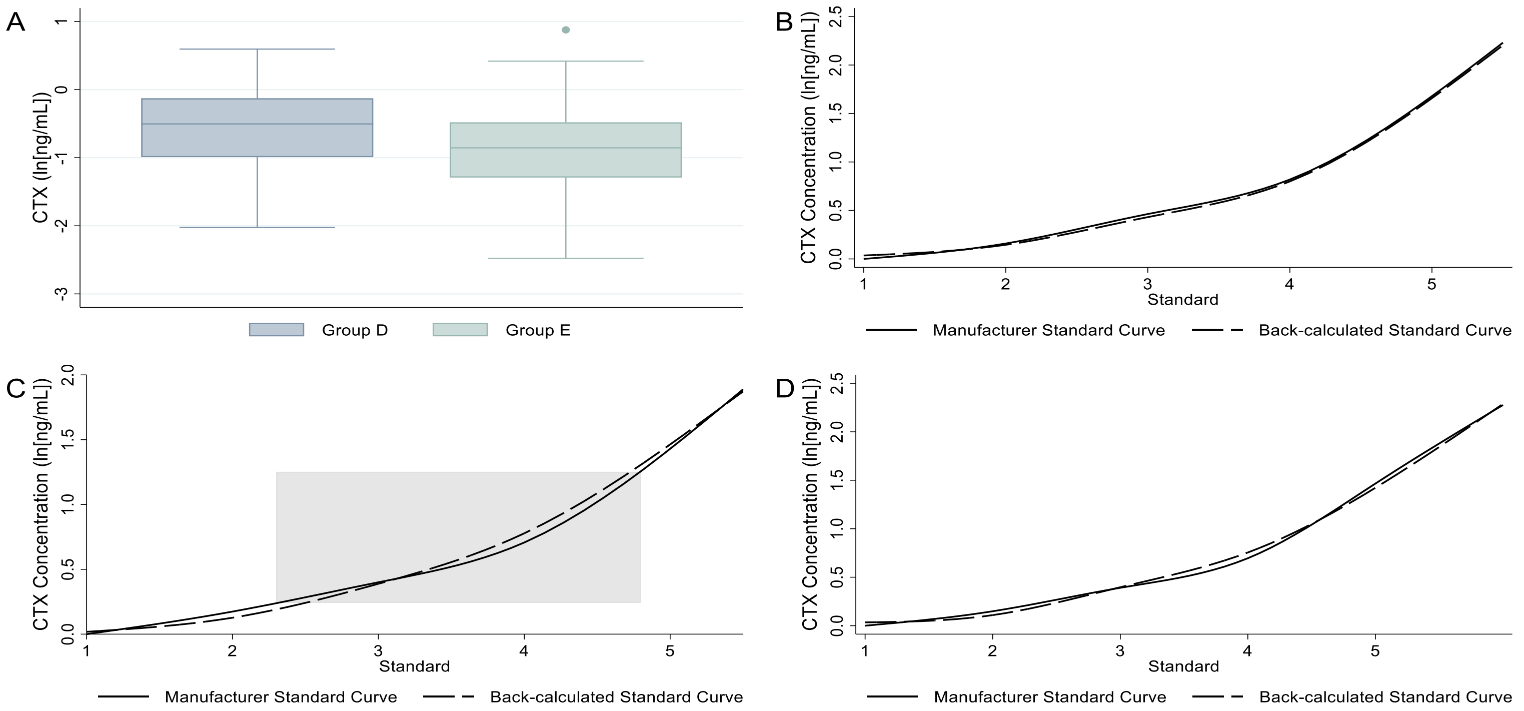

Supplemental Figure 3: Batch-related variation in CTx assay performance. CTx was measured in-house using a commercially-available ELISA kit. CTx values of women in group E were measured in the first assay batch and CTx values of women in group D were measured in the second assay batch. Boxplots show the distribution of delivery CTx concentrations differed between intervention group D and group E (panel ‘A’), even though participants in both groups were assigned the same prenatal vitamin D dose (28000 IU/week). The average back-calculated concentrations of each standard on the standard curve in the first batch corresponded closely to the manufacturer-labelled concentrations of each standard (panel ‘B’). However, in the second batch, there was clear systematic overestimation of CTx concentrations at values between standards 3 and 5 (panel ‘C’), which was the range within which the majority of CTx concentrations were observed, thereby accounting for the relatively higher average CTx group concentrations in group D vs E. The shaded gray area shows the 10th and 90th percentile of delivery and postpartum CTx concentrations in the second assay batch. CTx values measured in the third batch consisted of samples from women at enrolment (17-24 weeks’ gestation) and were equally distributed across intervention arms; back-calculated concentrations of the standard curve in the third batch generally matched the manufacturer-labelled concentrations, with slight overestimation of values at standard 4 (panel ‘D’). CTx, carboxy terminal telopeptide of type 1 collagen.

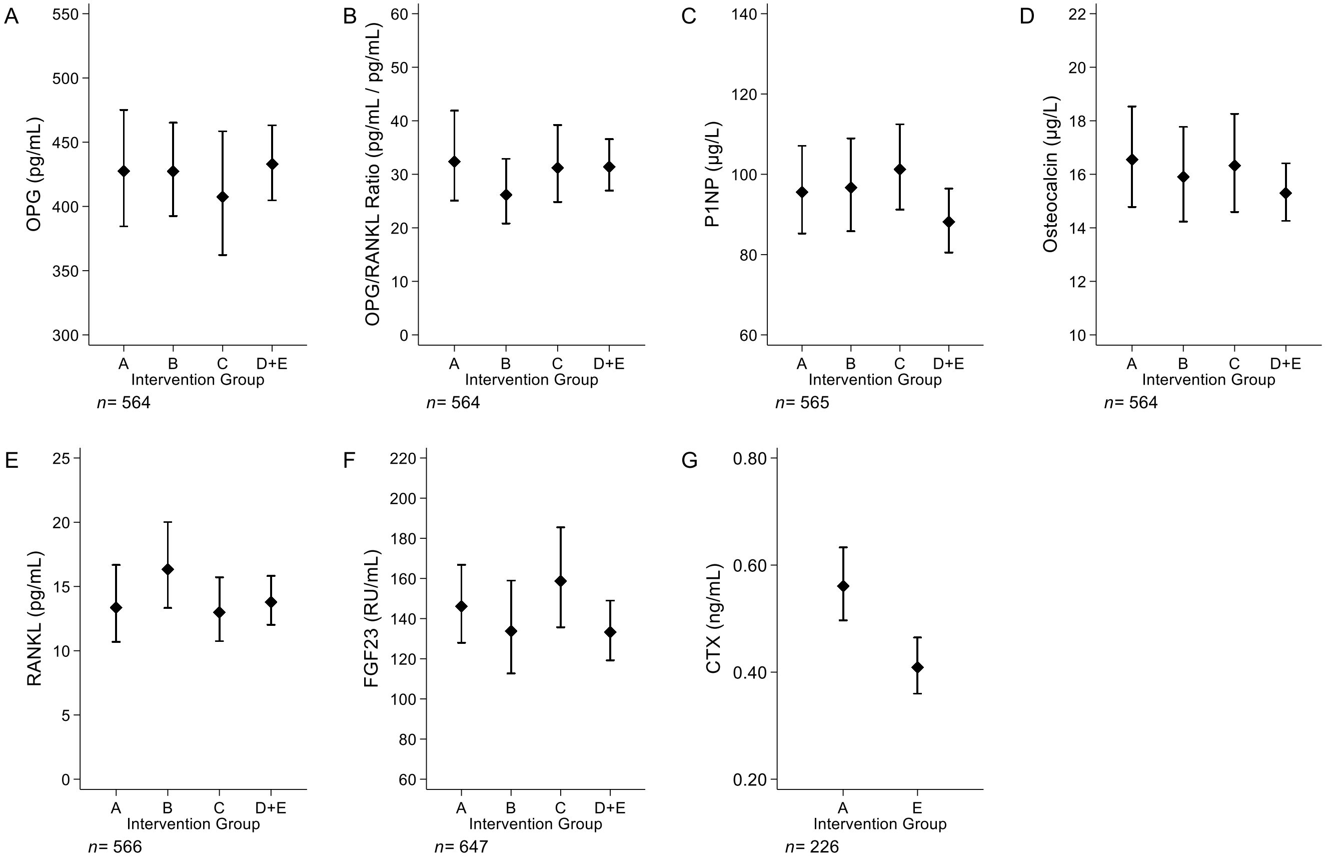

Supplemental Figure 4: Bone biomarker geometric mean, and 95% CI by vitamin D intervention group at delivery. Letters on the x-axis indicate the intervention groups that differed by prenatal;postpartum vitamin D doses (IU/week), as follows: A=placebo;placebo; B= 4200;placebo; C= 16800;placebo; D= 28000;placebo, E= 28000;28000. Groups D and E were combined due to equal prenatal dose except for CTX, which is presented for groups A and E only. Panel A: OPG, panel B: OPG/RANKL, panel C: P1NP, panel D: OC, panel E: RANKL, panel F: FGF23, and panel G: CTx. Differences in bone biomarker concentrations were estimated using linear regression of the log-transformed bone biomarkers on vitamin D intervention group. CTx concentration was significantly lower in intervention group E compared to group A; all other biomarkers did not significantly differ from placebo ($P>0.05$).

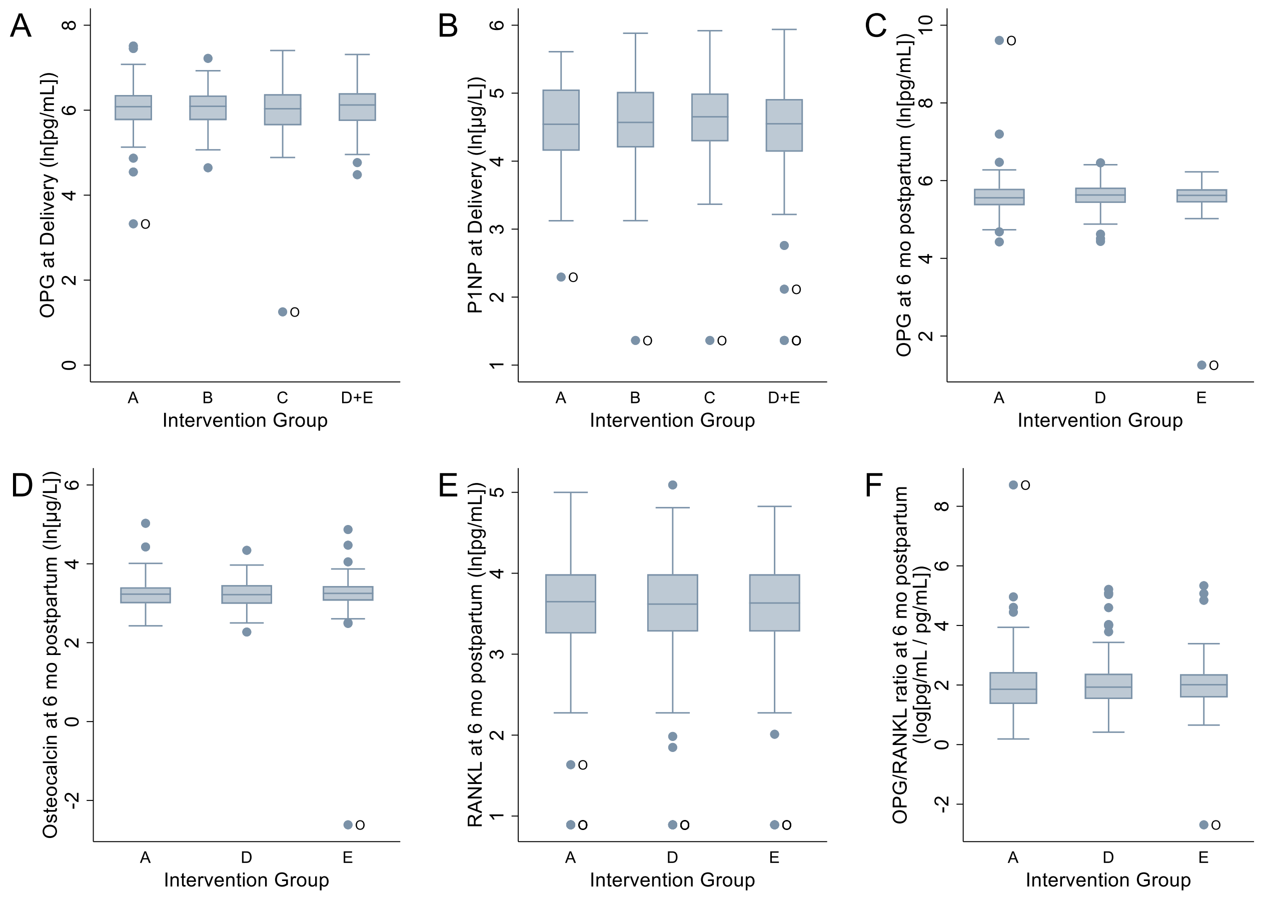

Supplemental Figure 5: Box plots showing the distribution of maternal bone biomarkers included in sensitivity analyses in which statistical outliers were removed. Data points labelled with an "O" were excluded in sensitivity analyses such that there were 2 data points excluded for OPG at delivery (panel 'A'), 7 data points excluded for P1NP at delivery (panel 'B'), 2 data points excluded for OPG at 6 months postpartum (panel 'C'), 1 data point excluded for osteocalcin at delivery (panel 'D'), 13 data points excluded for RANKL at 6 months postpartum (panel 'E'), and 2 data points excluded for the OPG/RANKL ratio at 6 months postpartum (panel 'F'). Intervention groups represent prenatal;postpartum vitamin D doses (IU/week) as follows: A=0;0, B=4200;0, C=16800;0, D=28000;0, E= 28000;28000 IU/week.

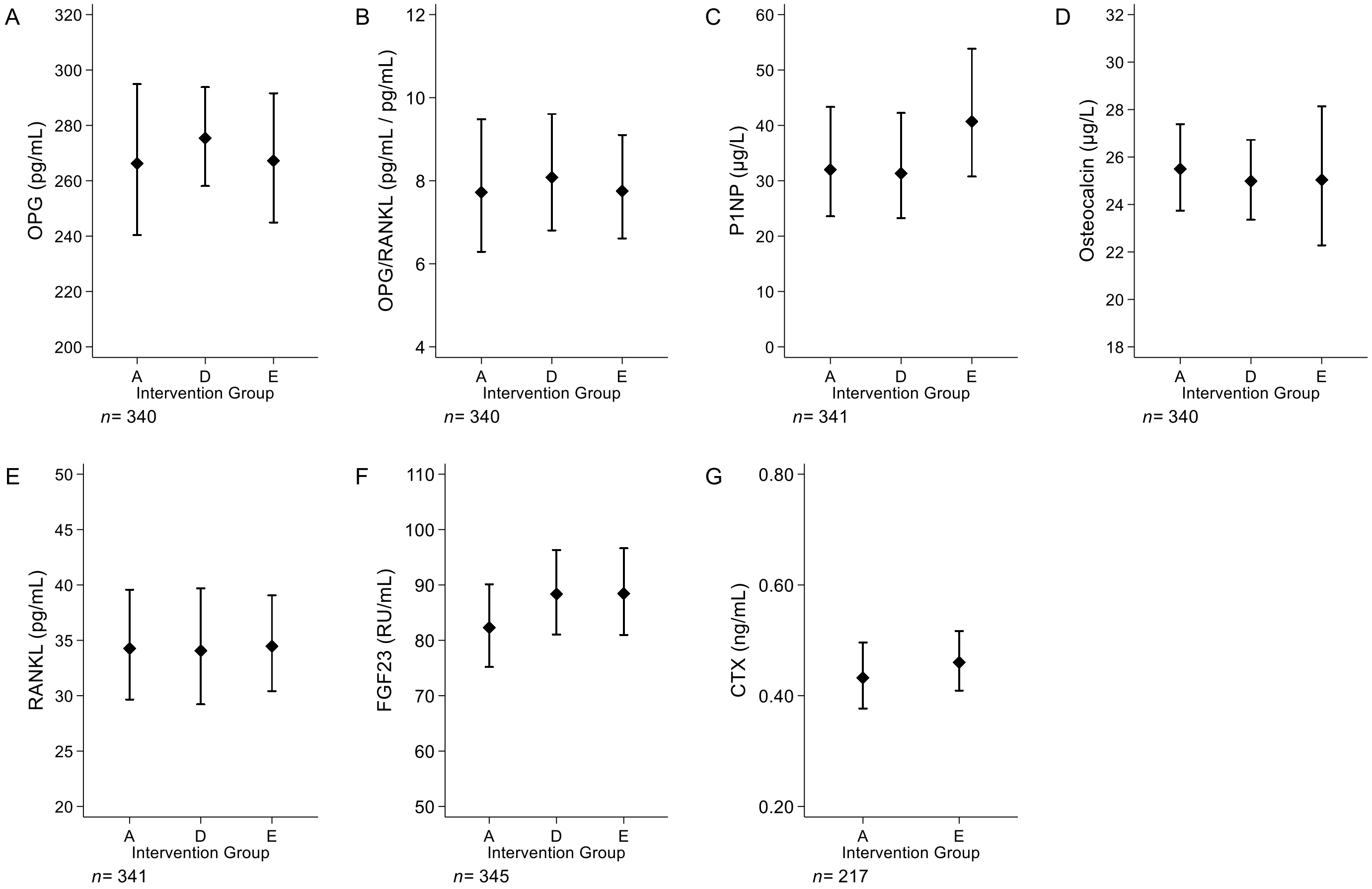

Supplemental Figure 6: Bone biomarker geometric mean, and 95% CI by vitamin D intervention group at 6 months postpartum. Letters on the x-axis indicate the treatment groups that differed by prenatal;postpartum vitamin D doses (IU/week), as follows: A=0;0; D= 28,000;0, E= 28,000;28,000. Panel A: OPG, panel B: OPG/RANKL, panel C: P1NP, panel D: OC, panel E: RANKL, panel F: FGF23, and panel G: CTx. The effect of vitamin D on bone biomarker concentrations were estimated using linear regression of each of the log-transformed bone biomarkers on vitamin D intervention group. Compared to placebo, bone biomarker concentrations did not significantly differ across the intervention groups

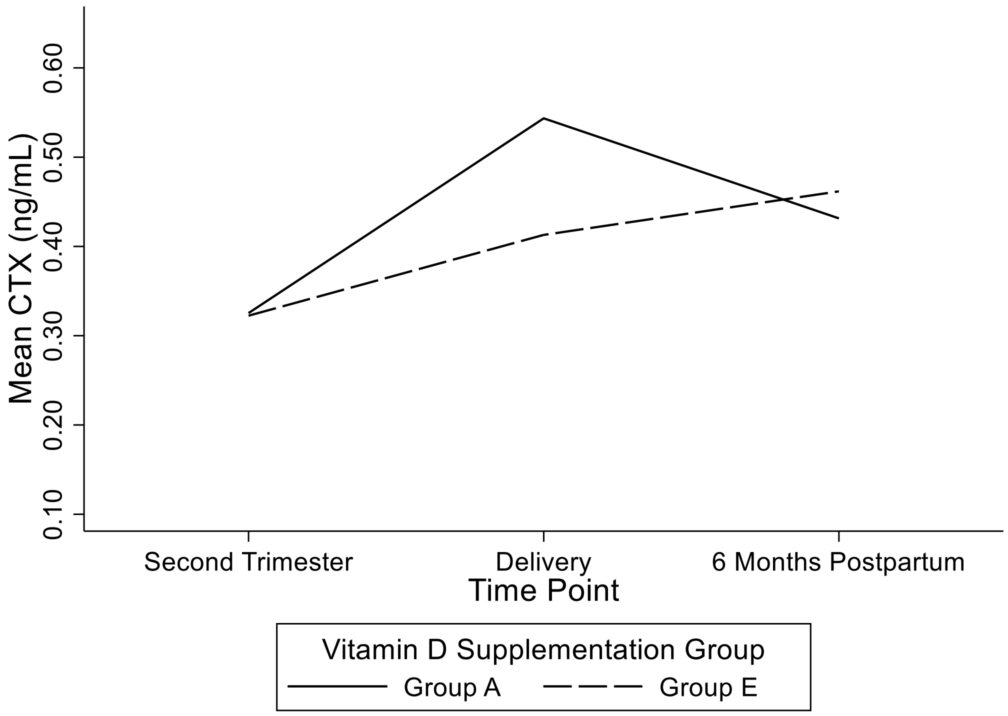

Supplemental Figure 7: Geometric mean CTx concentrations (ng/mL) of women who contributed biomarker data at enrolment (17-24 weeks' gestation), at delivery, and at 6 months postpartum, by vitamin D intervention group (*N*=209). Intervention groups represent prenatal;postpartum vitamin D doses (IU/week) as follows: A=0;0, E= 28000;28000 IU/week.

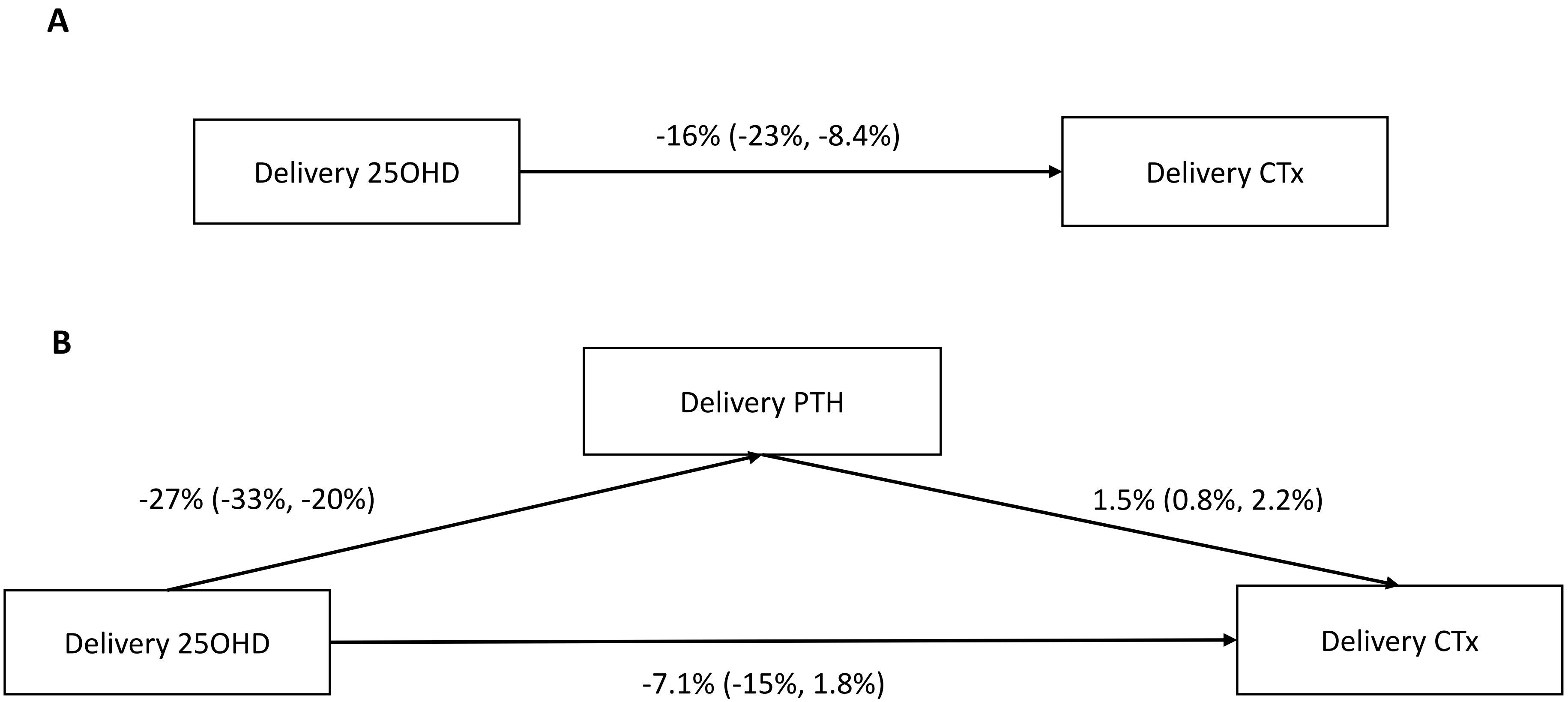

Supplemental Figure 8: The total effects (A) and direct and indirect effects (B) of maternal delivery 25-hydroxyvitamin D (25OHD) on CTx concentrations at delivery, mediated by parathyroid hormone (PTH) concentrations at delivery. Effect estimates represent the percent difference (95% confidence interval) in CTx concentrations for each 50 nmol/L increase in 25OHD or for each 10% increase in PTH.

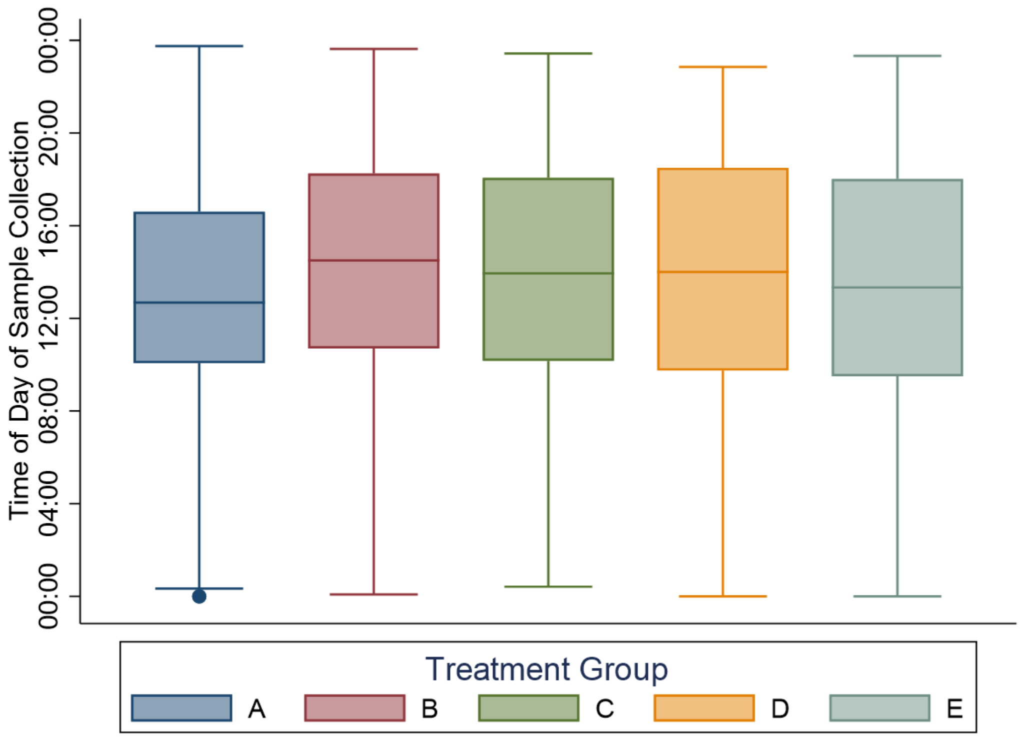

Supplemental Figure 9: Time of day of CTx sample collection, stratified by vitamin D treatment group (n=556). There was no significant difference in the timing of specimen collection at the delivery time point across treatment groups.). Intervention groups represent prenatal;postpartum vitamin D doses (IU/week) as follows: A=0;0, B=4200;0, C=16800;0, D=28000;0, E= 28000;28000 IU/week.
